# Multidisciplinary understanding of modifiable physical disability risk factors for health professionals: A scoping review protocol

**DOI:** 10.1371/journal.pone.0306438

**Published:** 2024-07-11

**Authors:** Derek Hanson, Stephen Samendinger, Edwin McCulley

**Affiliations:** 1 Nutrition Science & Wellness Department, State University of New York (SUNY)-Farmingdale, Farmingdale, NY, United States of America; 2 Health Sciences Department, Drexel University, Philadelphia, PA, United States of America; 3 Urban Health Collaborative, Philadelphia, PA, United States of America; 4 Department of Epidemiology and Biostatistics, Dornsife School of Public Health, Drexel University, Philadelphia, PA, United States of America; University of Ghana, GHANA

## Abstract

**Introduction:**

Physical disability represents a major burden to health and lifespan, particularly as the proportion of older adults within the United States is expected to grow. Prevention efforts for physical disability targets methods and strategies to decrease modifiable risk factors. Potential challenges for health professionals may exist in synthesizing and interpreting this broad spectrum of literature due to the discipline-specific nuance of language used, practice type or specialty, and lack of interdisciplinary resources. This scoping review will map and synthesize the literature across healthcare disciplines to identify modifiable risk factors and the evidence related to their ability to predict physical disability. We will also draw attention to the possibility of modifiable risk factors for physical disability being operationalized as pre-disability in order to strengthen primary and secondary prevention efforts.

**Methods:**

A planned search strategy using physical disability terminology will be searched in English across MEDLINE, CINAHL, Health Source, PEDro, and REHABDATA by two reviewers in line with our review objectives and inclusion criteria. Eligibility for inclusion include peer-reviewed primary research published in the English language and have established a relationship between a person-level measurable characteristic that is modifiable by changes in lifestyle behaviors and any of the commonly accepted terms used to categorize or describe physical disability.

**Expected results:**

Presentation of results will be using the PRISMA flowchart, with additional mapping and synthesis of evidence for modifiable risk factors for physical disability to clarify divergent terms used in classifying and measuring these factors and their potential for prediction of physical disability.

**Expected conclusion:**

It is anticipated that this scoping review will identify and highlight a variety of modifiable risk factors for physical disability that may aid primary and secondary prevention efforts for health professionals.

## Introduction

Physical disability (PD) represents a major burden to health and lifespan within the aging population, particularly as the number of older adults within the United States is expected to grow [1] Estimates of the prevalence of PD within the population highlight the magnitude of these public health problems, particularly in light of the increasing demographic trend. For example, a 2016 Morbidity and Mortality Weekly report summarized that 1 in 5 adults within the U.S. report a physical disability, including 26.9% of people who are aged 65 or older reporting a mobility disability [2]. The American Community Survey 1-year estimates of disability among people aged 65 and older in 2021 were 32.6% [3]. Awareness of prevention strategies of PD is therefore among continued importance and serves as the basis for professionals working to prevent or offset PD before its deleterious effects on a person’s health or lifespan occurs. Professionals across healthcare and health promotion aim to deliver these services using evidence-based practices, typically linked to the identification and screening of modifiable risk factors for physical disability (MrF-PD). Potential challenges for these professionals may exist in synthesizing and interpreting the broad spectrum of MrF-PD literature due to the discipline-specific nuance of language used, variances in screening tools, and lack of interdisciplinary resources. Additional inquiry into the complexities of these relationships may enhance the awareness of these challenges and the interdependent nature of many MrF-PD, as well as synthesize how MrF-PD may be integrated in each professional’s role in care.

### Disability language & disability measurement

Understanding commonly used disability language, standard methods of disability measurement, and recognizing the diversity in classifying disability level and type among these measures, is important in efforts to study MrF-PD. Attempts to standardize and enhance disability-related terms used by health professionals have been encouraged after the World Health Organization developed the International Classification of Functioning, Disability and Health (ICF) as a framework for defining health and disability [4]. As outlined in the *ICIDH-2*: *International Classification of Functioning*, *Disability and Health*, the ICF separates physical impairments related to disability by body function and structural level, the activity limitations that may result, as well as the participation restrictions related to the disability [4]. Additionally, as outlined in the ICIDH-2 report, distinctions within this classification model exist for Environmental and Personal factors that may be contributing factors, highlighting the key feature that disability is related to social, familial, and community-based activities that are typically part of the person’s behavioral norms. Since the ICF was created to represent the international standard for common language to be used across all disciplines when describing or measuring health and disability, collective uniformity in the understanding of these factors remains a question. As highlighted by Kostanjsek [5], the creation of the ICF attempting to standardize common disability language have theoretically further enabled public health organizations to track population rates of disability, inform professional care models in the evaluation, diagnoses, and treatment of patients, and guide prevention strategies toward increasing early awareness of disease signs and symptoms that are associated with modifiable risk factors.

While there exists some uniformity in disability language among common forms of measurement, how these terms are operationalized for type and level of disability in each measure can create some confusion. These differences can be particularly divergent in terms of reporting physical disability. In the United States, population-level disability data is primarily collected through the National Health and Nutrition Examination Survey (NHANES) [6], Behavioral Risk Factor Surveillance System (BRFSS) [7], and U.S. Census Bureau’s American Community Survey (ACS) [3]. NHANES includes a series of questions that measure a variety of health factors through in-person testing and/or telephone interviews. Questions related to disability are part of the “Functioning” series and focus on vision, hearing, cognitive function (anxiety and depression), communication, self-care, lifting a 2-liter bottle to chest height, mobility on even surfaces, stairs, and ability to go places in the environment for social functions. The BRFSS is an ongoing state-based telephone survey for non-institutionalized people 18 years of age and older. This survey includes questions on six disability types (hearing, cognition, vision, mobility, self-care, independent living). As part of the National Health Interview Survey (NHIS) [8], the ACS has collected information in six categories of disability in civilian non-institutionalized population: Hearing, Seeing, Cognitive, Mobility, Self-care, and Independent living. The ACS disability measure was originally designed to collect basic functioning data in these core domains, but also meant to identify the population at risk of disability. However, as of 2019, the NHIS has shifted to the use of Washington Group on Disability Statistics (WG) disability questions [9]. Using the ICF as a conceptual model, the WG set of disability questions were developed to improve international comparability of disability statistics, while identifying those at-risk of experiencing restricted social participation. The WG questions collect disability data from each of the same ACS domains except, instead of asking about independent living, the set of questions include the communication domain of functioning. The WG questions utilize an ordinal response format (no difficulty, some difficulty, a lot of difficulty, cannot do at all) in an attempt to gather more information about functional ability than typical dichotomous response options.

For this scoping review, we will further explore differences in disability language and measurement and how possible discrepancies may create challenges for researchers seeking to study factors associated with physical disability.

### Modifiable risk-factors & prediction of physical disability

Health professionals rely on a variety of conceptual models, tools, and science to prevent disease and disability. MrF-PD may be targeted at the primary or secondary prevention level. Primary prevention aims to prevent a disability or disease from ever occurring within healthy individuals, typically through limiting exposure to risks or boosting health defenses [10]. Secondary prevention efforts seek screening and early disease detection of subclinical forms of the disease that have yet to produce overt symptoms in *healthy-appearing* individuals [10]. In the context of PD, both of these prevention levels rely on the detection of objective characteristics that have been shown to increase the risk of subsequent PD, followed by the delivery of interventions or lifestyle modifications aimed at mitigating those risks. Of course, prevention models consider modifiable risk factors (MrF) those for which interventions or changes to a person’s lifestyle behaviors exist that can have a positive effect on the factor of interest. Many studies have investigated MrF-PD, including directly predicting future PD rates and demonstrating associations between PD and modifiable factors. Other studies have explored variables thought to be indirectly related to PD. For example, as a physical attribute that can be modified, gait speed has been correlated to future PD in a variety of studies [11–13]. Perhaps the most direct lifestyle-related modifiable risk factor, physical activity levels, have been linked to outcomes of physical function and future disability [14–18]. Furthermore, subjective characteristics have also been linked to future PD through the assessment of self-reported difficulties with physical function [19]. Increasing the complexity of these relationships further, is the fact that classifying various functional limitations as modifiable can be difficult if they may not be modifiable to a meaningful extent. There is also evidence to suggest that significant life events, such as hospitalization, are associated with PD [11, 20], but the antecedents to many such life events can’t always be predicted or classified as MrF.

Aside from smoking, as a distinct lifestyle behavior, four other common lifestyle-related health risk factors have been shown to be associated with disability: obesity, diabetes, high cholesterol, and hypertension [21]. To estimate the population-level disability prevalence related to these five modifiable risk factors, researchers studied data from the BRFSS and defined disability as global physical functioning, using self-reported responses on three physical functioning questions and excluding responses concerning vision-related and cognitive disability (16). Mehta and colleagues [21] classified these risk factors as modifiable lifestyle factors because each is influenced by diet and physical activity. However, along with the direct effects of these lifestyle factors, they are also known contributors to other disability-related risk factors (e.g., diabetes, high cholesterol, hypertension). We highlight this study, not only because it supports the association of lifestyle and PD, but it also speaks to the complexity of studying direct and indirect MrF-PD. The interdependent nature of these factors complicates what might be considered a direct or indirect MrF-PD suitable for prevention (versus treatment) strategies and how researchers measure the influence and outcomes of each. Nevertheless, health professionals delivering prevention services can benefit from increased awareness of the many direct and indirect MrF-PD that exist within the populations they are serving.

With abundant literature about the multifactorial nature of PD and its direct and indirect contributors, along with the fact that not all predictors of physical disability are modifiable risk factors and vice versa, we believe that an evidence-based synthesis of what is known is warranted. This scoping review will map the available evidence and report on the complexity in identifying multiple direct and indirect lifestyle-related risk factors as modifiable. Further, this scoping review will examine how this challenge of identifying MrF-PD affects their use in the prediction and prevention of physical disability. We will also attempt to organize common screening tools and functional assessments used in the measurement of these factors and evidence related to their usefulness in predicting physical disability.

## Objectives

The purpose of this scoping review is to systematically map and synthesize the literature across healthcare disciplines to identify modifiable risk factors and collate the evidence related to their ability to predict physical disability. In the process, we will bring clarity to the divergent terms used in classifying and measuring these factors and physical disability. We will also draw attention to the possibility of MrF-PD being operationalized as pre-disability in order to strengthen primary and secondary prevention efforts. Mapping and identifying key components of MrF-PD across the heterogeneous disciplines of healthcare and health promotion may also decrease confusion and redundancy, while addressing the gap in the literature that exists in bringing many of these related topics together.

A preliminary search for existing scoping and systematic reviews on the MrF-PD has been conducted during April 2023 using the PubMed database. While we did locate reviews restricted to specific healthcare disciplines such as Physical Therapy [22] seeking to identify predictors of disability, we are not aware of any existing review that matched our study objectives.

## Methods

### Design

Scoping reviews aim to map and characterize a body of literature, based on existing evidence. Early scoping review frameworks were developed by Arksey and O’Malley [23] and supplemented by Levac et al. [24]. Collectively, these frameworks are captured by the Joanna Briggs Institute (JBI) scoping review methodology [25]; which serves as the basis for our scoping review. The author group will be guided by the JBI scoping review framework, with results presented in accordance with the 2020 guidelines for reporting described in Preferred Reporting Items for Systematic Review and Meta-Analysis Extension for Scoping Reviews (PRISMA-ScR) [26]. While this scoping review protocol is ineligible for registration in PROSPERO, the scoping review will be registered in PROSPERO once this scoping review protocol has been finalized and prior to submission of the full scoping review for publication.

### Data selection

**Fig 1:**
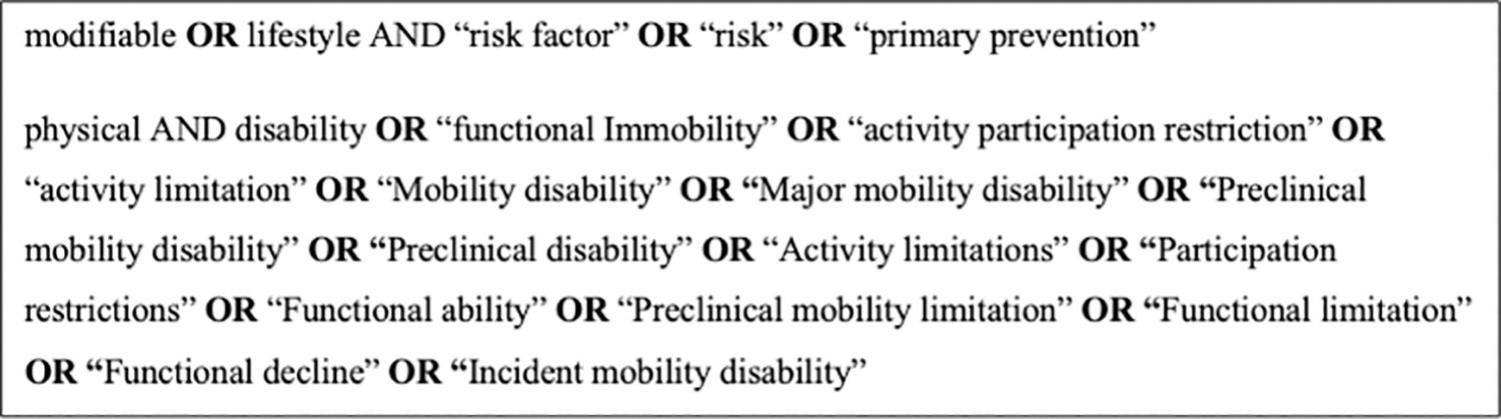
Database search initial key terms and Boolean strategy.

**Database searches.** Our database search will be carried out by two independent reviewers and will consist of a three-step strategy. The initial search will consist of two relevant databases (MEDLINE and CINAHL) utilizing the designated search terms, with the terms and boolean operators refined during the process (see Fig 1). Our second search will use the refined search across Health Source, PEDro, and REHABDATA. Our final search will attempt to identify additional relevant studies within the reference lists of the papers selected in previous searches.

#### Eligibility & inclusion criteria

Eligibility criteria for sources used will include peer-reviewed primary research published in the English language. Based on the objectives of our scoping review, the search strategy listed will target sources that have established a relationship between a person-level measurable characteristic that is modifiable by changes in lifestyle behaviors and any of the ICF terms used to categorize or describe PD. Studies will be excluded that include participants who have a diagnosis or disease state that is not related to or is not altered by lifestyle modifications. Although we will report on both objective and subjective measures, we will not restrict the search by screening for any specific or category of outcome measure.

### Data management

**Fig 2:**
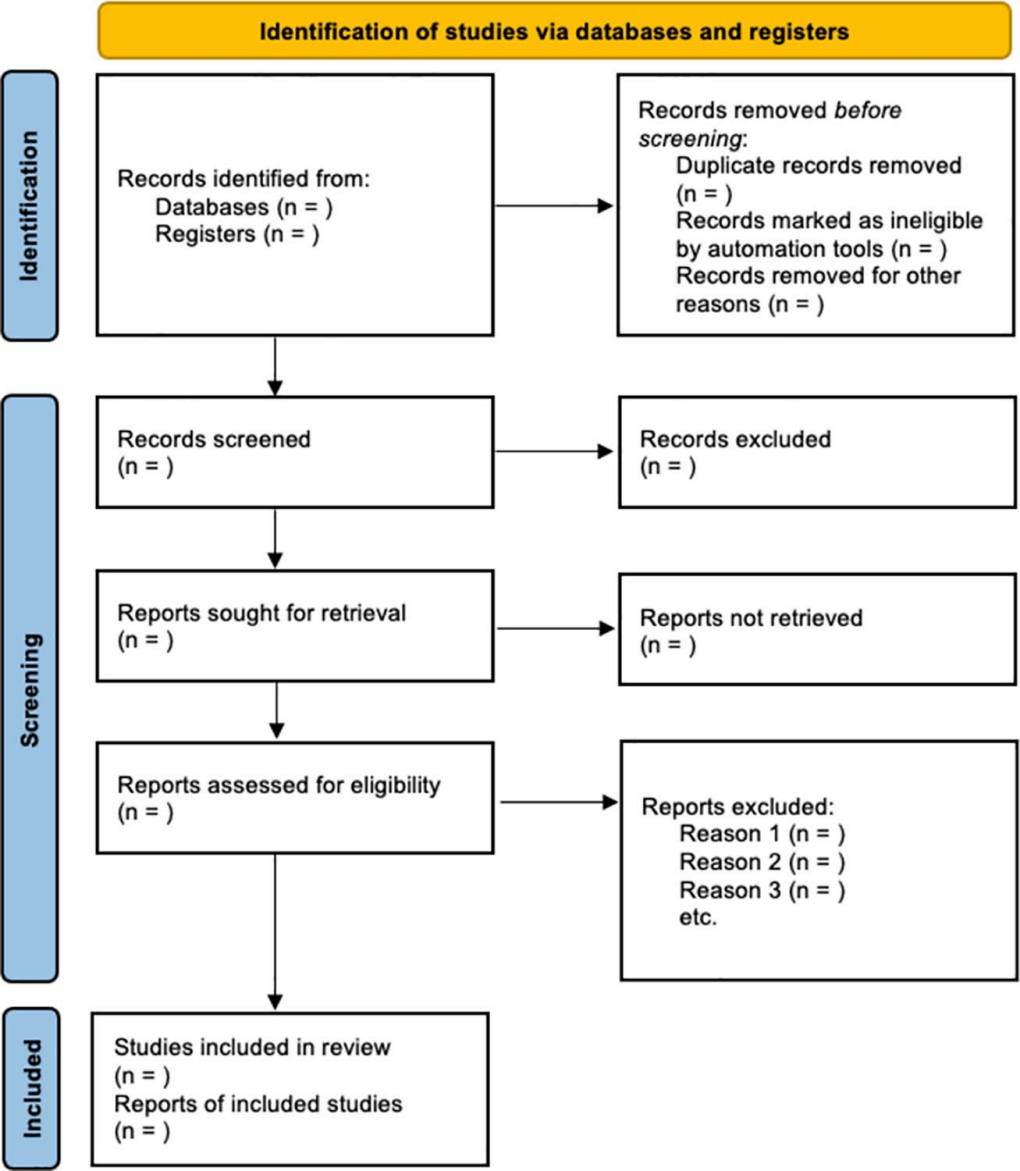
PRISMA flow diagram indicating identification, screening, inclusion and exclusion of studies via databases searched.

The process for selecting sources will be conducted after screening for duplicates from the various databases and includes: First level screening of article titles and abstracts for relevant inclusion criteria conducted independently by two researchers (i.e., without conferring with each other); similarly, an independent second level screening of all full-text articles that pass first level will be performed. Covidence systematic review software will be utilized throughout the screening processes. Any disagreements or inconsistencies in article selection will be resolved by consensus. Results from this screening and selection process will be documented and presented using the PRISMA flow diagram [27], as indicated in Fig 2.

### Data extraction items

Data from the articles will be coded for extraction based on study descriptive information (e.g., authors, year of publication) and alignment with the scoping review objectives and retrieved for review and synthesis. The exact number of variables for the extracted data will be refined as the review progresses but will include, at minimum: Author(s), Year of publication, Origin/country of origin (where the source was published or conducted), study Aims/purpose, Population and sample size, Methodology and Procedures, MrF-PD measurement type, Outcomes (including measures), Key findings that relate to the scoping review questions. We will also attempt to capture further study dynamics by extracting data related to *Study Design Population*: (I) Either individual-level, ecological (area-level), or experimental (not individual or ecological) or (II) *Study Design Time*: Either cross-sectional or longitudinal.

This scoping review will include descriptive findings, as well as a synthesis of the evidence related to the project’s objectives. Although this summary and synthesis will not be as extensive as conducted in a systematic review, we will attempt to describe predictive physical disability probabilities supported in this narrowed literature, carefully identify common MrF-PD variables in the literature, and identify gaps in the literature (e.g., quantifying, diagnosing, or characterizing MrF-PD).

### Data synthesis

In line with the heterogeneous nature of the MrF-PD, we plan to map and present the evidence found within our search through both tabular and descriptive format. To achieve this we will group the evidence in different ways, that can be based on preliminary searches, related to demographic considerations, physical characteristics, life events, and lifestyles. While each of these groups may represent some overlap with one another in respect to MrF-PD, we plan to map and report the strongest findings that most closely aligns with these categories, while also including any associations reported for any of these characteristics to provide additional support for these variables. Further, we plan to provide a summary analysis for quantitative data and thematic analysis for qualitative data, while pinpointing any gaps in the literature. Data abstraction will be standardized based on the above criteria, and conducted independently by two reviewers, with disputes handled via consensus with a third reviewer.

## Discussion

This interdisciplinary work will provide a map and synthesis of evidence for modifiable risk factors for physical disability to clarify divergent terms used in classifying and measuring these factors and their potential for prediction of physical disability. While the scope of PD affecting the population may be too large for a single scoping review and represents a burdensome limitation for our work, we believe that there is sufficient literature available to begin to synthesize some common understanding towards our objective. Through highlighting and identifying key components of MrF-PD across the heterogeneous disciplines of healthcare, while summarizing the evidence across a complicated and evolving body of evidence, we believe that this will serve to strengthen primary and secondary prevention efforts across these disciplines. Primarily, through the presentation and discussion of each of the MrF-PD for health professionals across these disciplines to enhance their understanding of these factors that they may have strategies to address with all appropriate stakeholders. Finally, this review intends to strengthen the concept of pre-disability, the idea that there is a period of time of subclinical changes associated with physical disability that are detectable, so that researchers and health-practitioners can continue to work towards the improvement of health for people who are at risk these potentially negative outcomes.
